# Supplementary material for: Widening area-based socioeconomic inequalities in cancer mortality in Germany between 2003 and 2019
Source: Sci Rep. 2023 Oct 19;13:17833. doi: 10.1038/s41598-023-45254-5 (PMC10587166; doi:10.1038/s41598-023-45254-5)
Supplement: Supplementary file 1 — Supplementary Information. [file 41598_2023_45254_MOESM1_ESM.pdf]

Supplemental Material

Widening area-based socioeconomic inequalities in cancer mortality in Germany between 2003 and 2019

Fabian Tetzlaff[1]*, Enno Nowossadeck[1], Lina Jansen[2], Niels Michalski[1], Ben Barnes[3], Klaus Kraywinkel[3], Jens Hoebel[1]

[1]Division of Social Determinants of Health, Department of Epidemiology and Health Monitoring, Robert Koch-Institute, Berlin, Germany

[2]Epidemiological Cancer Registry Baden-Württemberg, German Cancer Research Center (DKFZ), Heidelberg, Germany

[3]German Centre for Cancer Registry Data, Department of Epidemiology and Health Monitoring, Robert Koch-Institute, Berlin, Germany

***Corresponding author**

Fabian Tetzlaff
Robert Koch-Institut
FG28 Social Determinants of Health
Nordufer 20
13302 Berlin
Germany
TetzlaffF@rki.de

Table S1 Definition of total cancer and site-specific cancers according to ICD-10GM classification

total cancer	C00-C97, without C44, C77-79
oral and upper respiratory tract	C00-06, C09-14, C32
oesophagus	C15
stomach	C16
colon	C18-20
liver	C22
pancreas	C25
lung	C33-34
malignant melanoma of skin	C43
(female) breast	C50
cervix uteri	C53
ovary	C56
prostate	C61
kidney	C64
bladder	C67
lymphoid and hematopoietic neoplasms	C81–96

Table S2 Additional descriptive information on the study population within the quintiles of socioeconomic deprivation between 2003 and 2019

		Number of Districts	Mean Population in Districts	Standard deviation	Min	Max
Men	–Deprivation quintile 1 (least deprived)	81	122.722	122.089	19.809	863.204
	–Deprivation quintile 2	80	100.912	89.606	19.055	549.779
	–Deprivation quintile 3	80	95.660	58.068	19.320	271.394
	–Deprivation quintile 4	80	104.136	188.525	16.961	1.692.432
	–Deprivation quintile 5 (most deprived)	80	77.381	46.110	19.984	285.557
Women	–Deprivation quintile 1 (least deprived)	81	127.374	128.600	21.563	906.913
	–Deprivation quintile 2	80	105.118	94.834	20.342	582.366
	–Deprivation quintile 3	80	98.932	60.123	21.285	283.057
	–Deprivation quintile 4	80	108.488	197.077	17.552	1.766.505
	–Deprivation quintile 5 (most deprived)	80	80.131	48.271	21.330	298.073

Figure S1 Time trend in age-standardised mortality rate by sex and regional socioeconomic deprivation

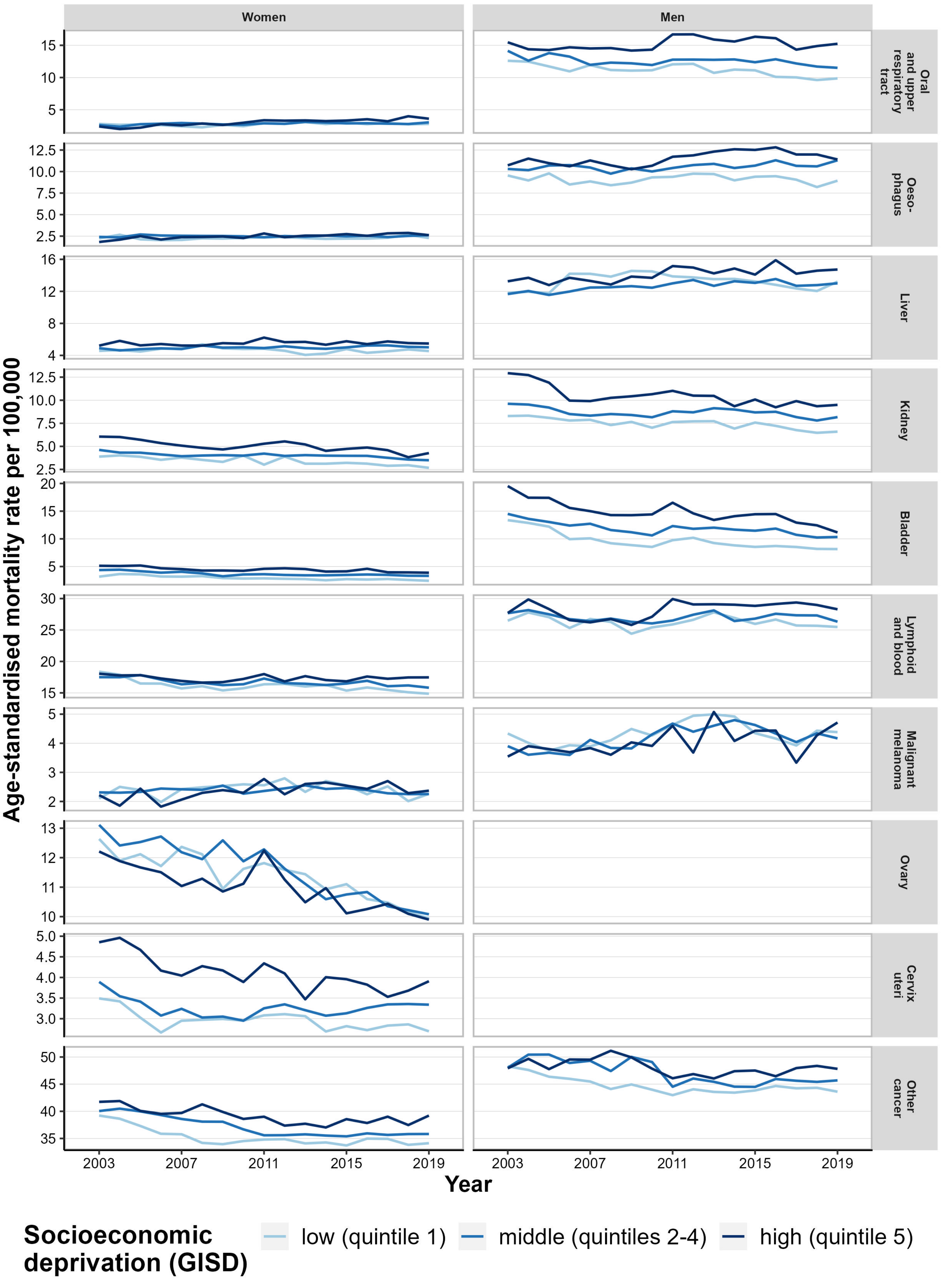

Figure S2 Absolute (SII) and relative (RII) inequalities in cancer site-specific Mortality by sex and year

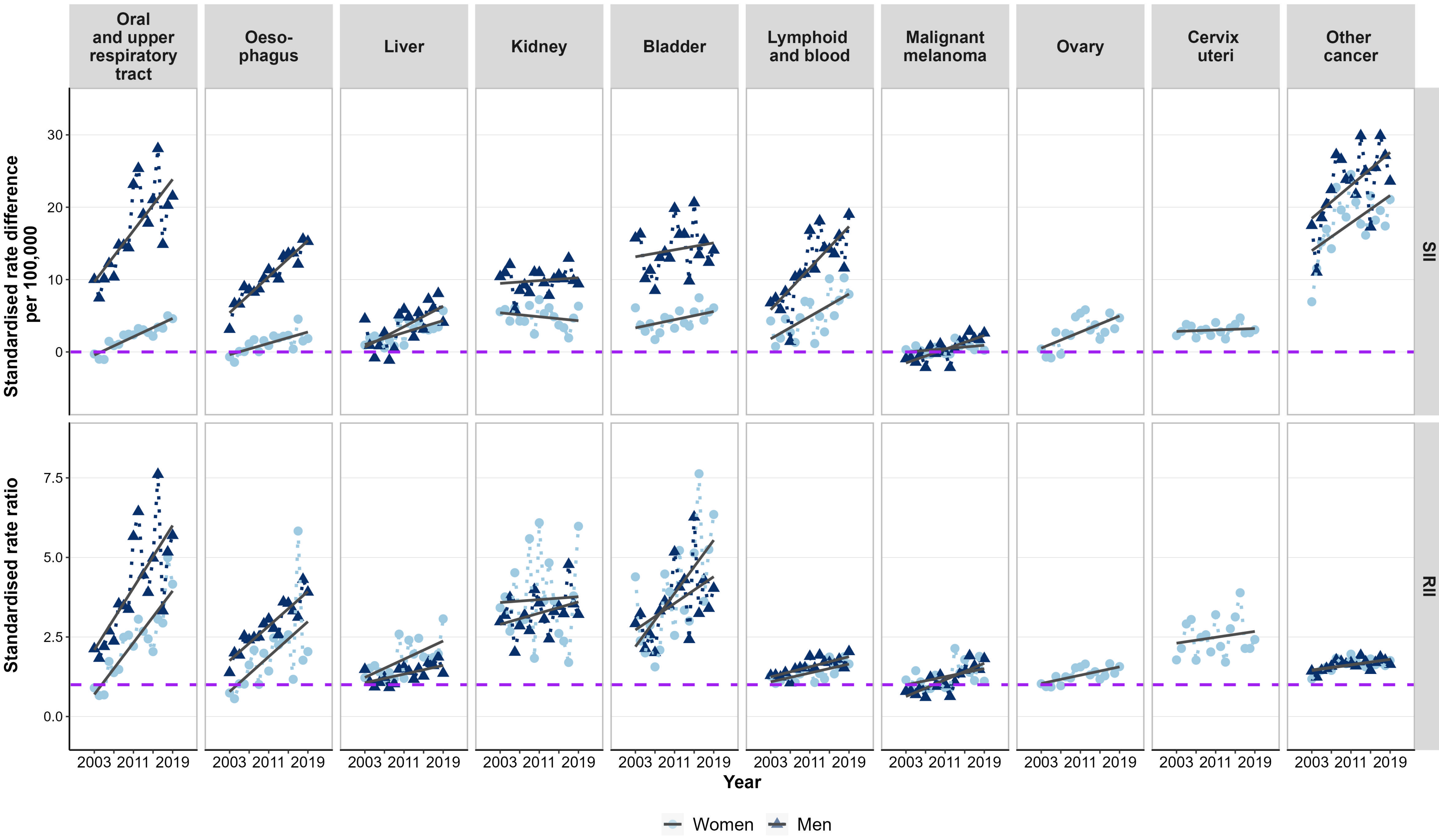

Note: SII, Slope Index of Inequality; RII, Relative Index of Inequality
